# Mass spectrometry data and size exclusion chromatography profiles of Australian taipan venom toxins

**DOI:** 10.1016/j.dib.2016.09.005

**Published:** 2016-09-14

**Authors:** Julian A. Harrison, J. Andrew Aquilina

**Affiliations:** a School of Biological Sciences, Faculty of Science, Medicine and Health, University of Wollongong, Australia; b Illawarra Health and Medical Research Institute, Northfields Avenue, Wollongong, NSW 2522, Australia

## Abstract

The compositions of paradoxin and taipoxin (PDx and TPx, respectively) were investigated using size exclusion chromatography (SEC) and nano-electrospray ionization mass spectrometry (nano-ESI-MS). The elution profiles from size exclusion chromatography of the venoms from *Oxyuranus microlepidotus* and *Oxyuranus scutellatus* were similar. Fractions corresponding to the trimeric toxins were treated with guanidinium hydrochloride and the individual subunits were separated by HPLC. In this report we present the size exclusion chromatography profiles for these toxins, and the nano-ESI mass spectra of the subunits after separation by HPLC: the first such comparative study of these toxins at the protein level. Data in this article are associated with the research article published in *Toxicon*: “Insight into the subunit arrangement and diversity of paradoxin and taipoxin” (J.A. Harrison, J.A. Aquilina, 2016) [1].

**Specifications Table**

**Table:** 

Subject area	*Chemistry and Biology*
More specific subject area	*Structural proteomics*
Type of data	*Table, figure*
How data was acquired	*Size exclusion chromatography, HPLC and mass spectrometry*
Data format	*Analyzed*
Experimental factors	*Amino acid sequences of toxin subunits were sourced from uniprot.org*
Experimental features	*Toxin oligomers and subunits were separated by chromatographic techniques for analysis by mass spectrometry*
Data source location	*University of Wollongong, NSW, Australia*
Data accessibility	*Data are within this article*

**Value of the data**

• Outlines the purification steps required to separate oligomeric PLA_2_.
• Presents size exclusion chromatography elution profiles for comparison with oligomeric toxins from other snake species.
• Describes isolation of individual toxin subunits for further characterization such as for sequence and post-translational modification analysis.
• Describes the instrumentation and conditions required for acquiring ESI mass spectra of the oligomeric toxins and subunits.
• Presents nano-ESI mass spectra of oligomeric toxins and subunits from *Oxyuranus microlepidotus* and *Oxyuranus scutellatus*. These spectra can serve as a comparison for those from other species.

## 1 Data

Three figures showing the size exclusion chromatograms for whole venom analysis and the nano-ESI mass spectra of the peaks from the HPLC of PDx and TPx subunits. Three tables, two containing the experimental subunit masses determined using nano-ESI-MS after HPLC separation, and a third showing the sequence data for the previously characterized subunits of TPx and PDx.

## 2 Experimental design, materials and methods

### 2.1 Size exclusion chromatography (SEC)

**Fig. 1:**
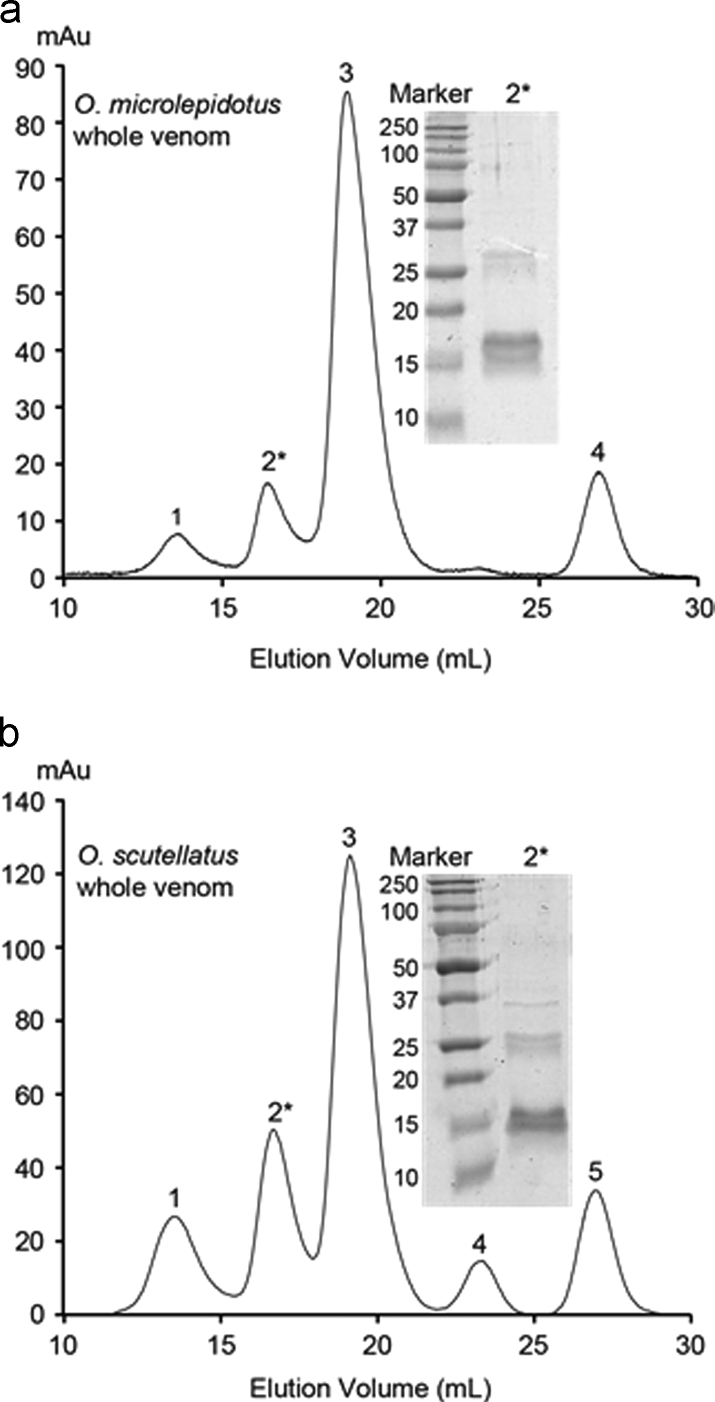
Separation of ~45 kDa toxins from *Oxyuranus* spp. whole venom via SEC. Chromatograms show the constituents of *O. microlepidotus* (A) and *O. scutellatus* (B) whole venom which where partitioned by SEC. Each of the individual SEC peaks were numbered in order of elution, and those peaks previously reported to contain PDx and TPx are marked with an asterisk [2], [3], [4]. The molecular weight of each marker band is given in kDa.

For each of the chromatographic runs, lyophilized whole venom from *O. microlepidotus* or *O. scutellatus* was dissolved in 200 mM ammonium acetate (NH_4_OAc) and loaded onto a Superdex 100 10/30 size exclusion column (GE Healthcare, Uppsala, Sweden). Fig. 1 shows the elution profiles. The second fractions (labeled 2, primarily containing the trimeric PLA_2_, PDx or TPx) were analyzed by SDS-PAGE (Fig. 1, *insets*). Each gel consisted of a 4% stacking gel and 15% resolving gel. Electrophoresis was performed using a Mini Protean 3 system (Bio-Rad, California, U.S.A), which was powered by a Bio-Rad power pack 300 power supply (Bio-Rad, California, U.S.A). Protein samples were mixed with a cracking buffer solution (0.5 M Tris–HCl, pH 6.8: 14 mL, 0.02 g Coomassie Blue, glycerol: 16 mL, 10% SDS: 16 mL) at a ratio of 1:1, then heated to 100 °C for 5 min prior to loading 10 µL of each sample into individual wells. Molecular weight standards were 5 µL of Precision Plus Protein™ Dual Color Standards (Bio-Rad, California, U.S.A.). Separation was achieved at 120 V after 1 h. Gels were stained with Coomassie Blue for at least 2 h, followed by destaining until protein bands were visible. Gels were scanned using a GS-800 calibrated densitometer (Bio-Rad, California, U.S.A).

### 2.2 High pressure liquid chromatography (HPLC)

To separate individual subunits, PDx and TPx were denatured in a solution of 6 M guanidinium hydrochloride (GuHCl) for 1 h then centrifuged for 10 min at 16,000*g* in a SIGMA 1-14 microfuge (SciQuip, Shrewsbury, UK). A 100 µL aliquot of each solution was loaded onto a Jupiter 5 μm C18 300 Å (150×4.6 mm) reverse-phase chromatographic column (Phenomenex, California, USA) equilibrated with Solution A (0.085% TFA in water (v/v)), using a Shimadzu VP Series HPLC System (Kyoto, Japan). Fractions were eluted (0.5 mL/min) using an acetonitrile gradient: 5–35% solution B over 20 min, 35–45% B over 40 min, 45–95% B over 20 min (Solution B: 0.085% TFA in acetonitrile (v/v)). HPLC fractions were lyophilized and stored at −20 °C until required.

### 2.3 Mass spectrometry analysis of HPLC samples

**Fig. 2:**
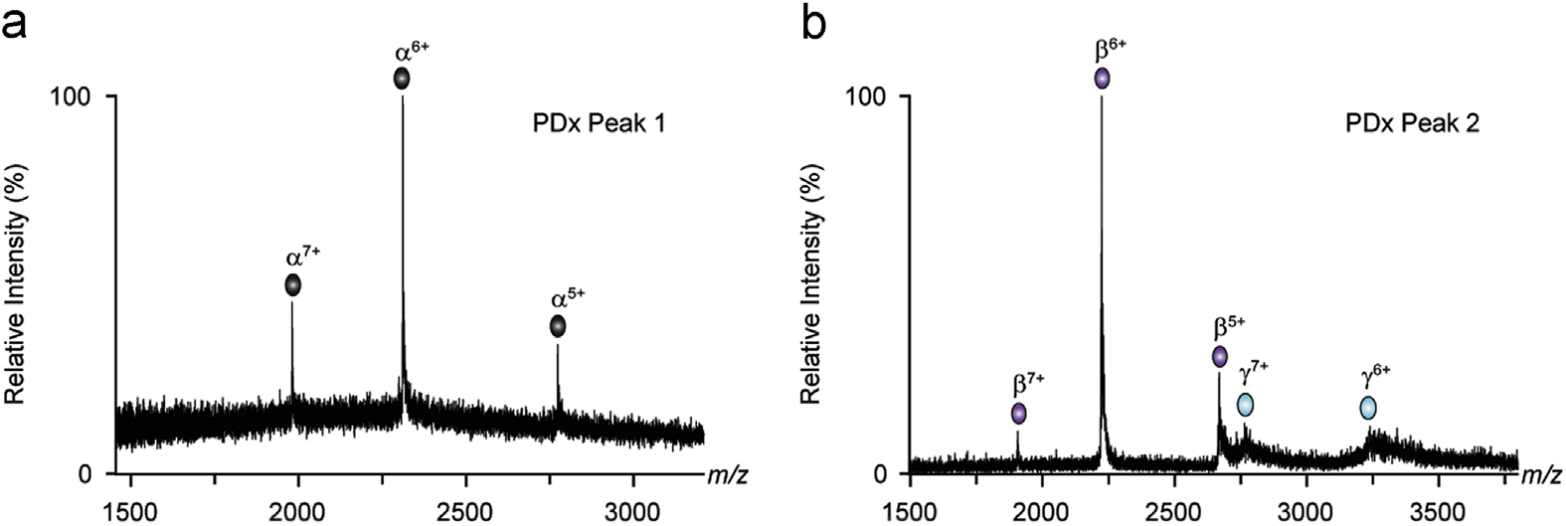
Mass spectra of PDx subunits separated by HPLC [1]. Spectra exhibit multiple charge states. The spectrum from the first HPLC peak (Figure 5 in [1]) for PDx (a) shows a charge state series between *m/z* 1750 and 2800 while the second HPLC peak (Figure 5 in [1]), (b) shows two series of charge states between *m/z* 1750 and 3350. The subunit identity of each series is shown above the peaks (α, β, and γ).

**Fig. 3:**
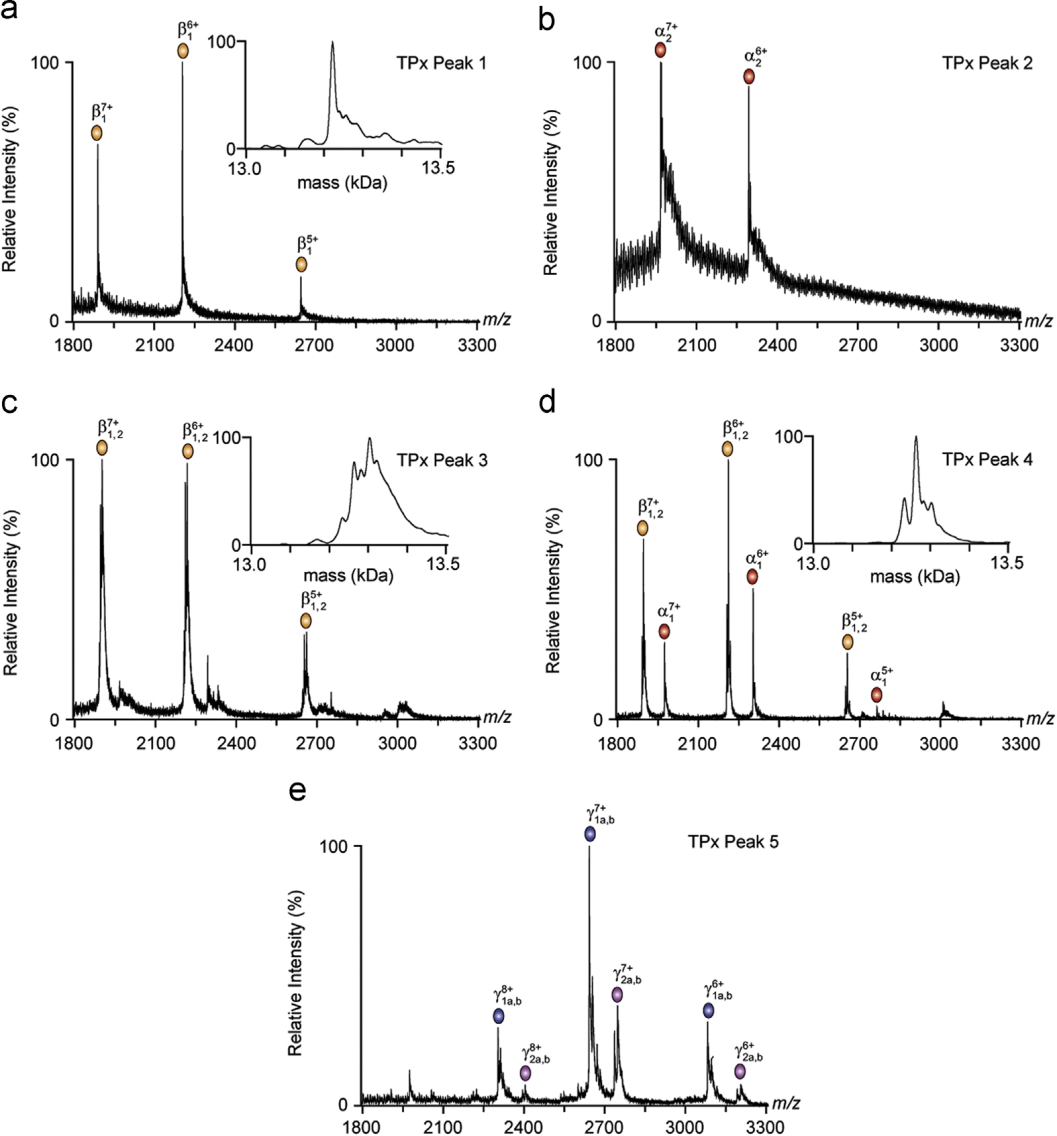
Mass spectra of TPx subunits separated by HPLC [1]. For HPLC peaks one to four (Fig. 5 in [1]), charge state series were evident between 1800 and 2750 *m/z*, while peak 5 (Figure 5 in [1]) contained components with charge states arising within a higher *m/z* window. Each inset shows the mass distribution of β_TPx_ isoforms for relevant HPLC peaks. The subunit identified by each series is shown on the top of each spectra.

All spectra were acquired using a Synapt (G1) HDMS time-of-flight mass spectrometer (Waters, Manchester, UK) and calibrated against a spectrum of CsI solution (10 mg/mL) acquired on the same day. Samples from HPLC separations were dissolved in 200 mM NH_4_OAc and introduced into the vacuum region of the instrument by electrospraying 2 µL of solution from a gold-coated borosilicate nano-ESI capillary (prepared in-house). Conditions for the acquisition of mass spectra were as follows: capillary voltage, 1.5 kV; sample cone, 140 V; extraction cone, 4 V; trap collision 12.5 V; transfer collision 25 V; collision cell gas pressure, 1.16×10^−2^ mbar; backing gas, 4.02×10° mbar. Mass spectra of the subunits of the toxins are shown in Fig. 2, Fig. 3; the masses determined from the spectra are shown in Table 1, Table 2. These data were then compared to the existing sequence data for these toxins (Table 3).

## Figures and Tables

**Table 1:** Nano-ESI-MS analysis of PDx components separated using HPLC. The number of each HPLC peak (Fig. 5 in [1] and Fig. 2 above) is shown in the left column of the table. The right column shows the observed mass of each protein detected in the nano-ESI-MS analyses, with the proposed identity in brackets.

**Peak**	**Masses (Da) and Identity**
1	13 862 (α)
2	13 318 (β)
	19 346 (γ)

**Table 2:** Nano-ESI-MS analysis of TPx components separated using HPLC. The number of each HPLC peak (Fig. 5 in [1] and Fig. 3 above) is shown in the left column of the table. The central column shows the observed mass of each protein detected in the nano-ESI-MS analyses, with the proposed identity in brackets. On the right, possible modifications to the disulfide linkages are proposed to account for differences between our findings and data in the published literature [4], [5], [6], [7].

**Peak**	**Masses (Da) and Identity**	**[S−S]***_**n**_*
1	13 222 (β_1_)	7
2	13 760 (α_2_)	7
3, 4	13 264 (β_1_)	6 + [Na^+^]_2_
	13 303 (β_2_)	5 + [H^+^]_4_
	13 232 (β_1_)	2 + [H^+^]_10_
5	18 492 (γ_1_*a*)	
	18 572 (γ_1_*b*)	
	19 149 (γ_2_*a*)	
	19 229 (γ_2_*b*)	

**Table 3:** Previously published amino acid sequences of PDx and TPx subunits [4], [5], [6], [7], [8]. Table shows the identity of the subunits that were detected using mass spectrometry. The left most column shows the subunit, the columns to the right show the subunit sequence and mass (sequence summed and with cysteine bonds intact) respectively.

**Subunit**	**Amino Acid Sequence**	**Mass (Da)**
PDx β	NLLQFGFMIECAIRNRQPALDFMNYGCYCGTVGHGTPVDDLDRCCKTRNECYAEAEKHGCYPSLTTYRWQCGRVGLHCNSKTQCEVFVCACDLAAAKCLAQEDYNPAHFNINTKARCR[8]	Sequence: 13332
		Cys bonds: 13318
TPx α	NLLQFGFMIRCANRRSRPVWHYMDYGCYCGKGGSGTPVDDLDRCCQVHDECYGEAVRRFGCAPYWTLYSWKCYGKAPTCNTKTRCQRFVCRCDAKAAECFARSPYQNSNWNINTKARCR[6]	Sequence: 13829
		Cys bonds: 13815
TPx β_1_	NLVQFGKMIECAIRNRRPALDFMNYGCYCGKGGSGTPVDDLDRCCQVHDECYAEAEKHGCYPSLTTYTWECRQVGPYCNSKTQCEVFVCACDFAAAKCFAQEDYNPAHSNINTGERCK[4], [7]	Sequence: 13236
		Cys bonds: 13222
TPx β_2_	NLVQFGFMIECAIRNRRPALDFMNYGCYCGTVGRGTPVDDLDRCCQVHDECYATAEKHGCYPSLTTYQWECRQVGNECNSKTQCEVFVCACDLAAAKCLAQEDYNPAHFNINTGERCK[4]	Sequence: 13313
		Cys bonds: 13229
TPx γ	SEIPQPSLDFEQFSNMIQCTIPCGESCLAYMDYGCYCGPGGSGTPIDDLDRCCKTHDECYAEAGKLSACKSVLSEPNNDTYSYECNEGQLTCNDDNDECKAFICNCDRTAVTCFAGAPYNDLNYNIGMIEHCK[5]	Sequence: 14602
		Cys bonds: 14588
